# Public interest in drug-related problems reflected in information search trends: an infodemiological study

**DOI:** 10.1007/s40199-024-00519-w

**Published:** 2024-06-18

**Authors:** Laura Martínez-Aguilar, María Sanz-Lorente, Fernando Martínez-Martínez, María J. Faus, Javier Sanz-Valero

**Affiliations:** 1 Pharmaceutical Research Group of the University of Granada, Faculty of Pharmacy, University of Granada, Granada, Spain; 2 Center of Public Health, Consellería of Universal Health and Public Health, Valencia, Manises, Spain; 3 Carlos III Health Institute, National School of Occupational Medicine, Madrid, Spain

**Keywords:** Access to information, Internet access, Information-seeking behaviour, Medication errors, Drug interactions, Drug overdose, Drug-related side effects and adverse reactions, Contraindications, drug

## Abstract

**Background:**

The analysis of how people search and “navigate” the internet to obtain health-related information and how they communicate and share this information can provide valuable knowledge about the disease patterns behaviour and health habits of populations.

**Objective:**

To determine the population’s interest in drug-related problems through information search trends.

**Method:**

A descriptive ecological correlational study, based on obtaining Google Trends data. Variables studied: relative search volume (RSV), evolution over time, milestones and seasonality.

**Results:**

The most searched topic was *drug overdose*, with mean RSV of 56.25 ± 0.65. The highest increase occurred in the *contraindication* topic (R^2^ = 0.87, *p <* 0.001). The main milestone was observed in the *drug overdose* topic in July 2018 (RSV = 100). A very close relationship was found between *adverse drug reaction* and *contraindication* (R = 0.89, *p <* 0.001). Slight seasonality was noted in the *adverse drug reaction* (augmented Dickey–Fuller test [ADF] = −1.96), *contraindication* (ADF = −2.66) and *drug interaction* (ADF = −1.67) topics, but did not show an epidemiological trend.

**Conclusions:**

The greatest public interest was found in the *drug overdose* and *contraindication* topics, which showed a stronger upward trend, although the seasonality study did not show any very notable data or demonstrate epidemiological information search behaviour. The main milestone observed was due to media factors related to the consumption of narcotics. There was a clear difference in English-speaking countries in the use of the *drug overdose* topic. A correlation between the *adverse drug reaction* and *contraindication* topics was confirmed.

**Graphical Abstract:**

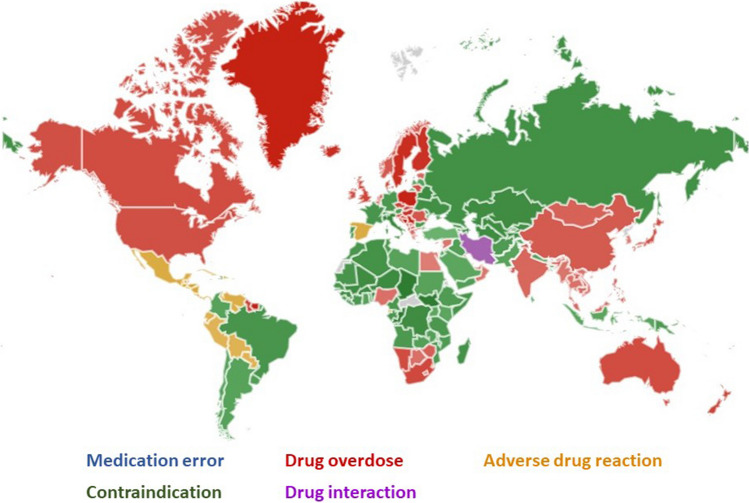

## Introduction

For years one of the objects of study in the field of pharmaceutical care has been so-called drug-related problems (DRPs), a controversial term much discussed among health professionals themselves.

The intense debate on the suitability and meaning of the term DRP is reflected in the great variety of designations used in the scientific articles that have been published on this subject and probably explains the absence of a word (descriptor) that uniquely identifies this concept [1]. These discrepancies, maintained over time, with respect to the definition and classification of DRPs has led the Pharmaceutical Care Research Group at the University of Granada to present several consensus studies at various stages [1–3].

These differences of terminology are not easy to explain and could be due to the different terms used by authors in their publications [4]. It is therefore not surprising that the review by Pintor-Mármol et al. [5], identified 60 terms for referring to medication-related patient safety with 189 different definitions. Obviously, this is reflected in the scientific literature on DRPs and it should not surprise us that this polysemous usage is shared by the general public. Similarly, it is natural that this situation should also apply when searching for information on the Internet.

The absence of a descriptor to standardize indexing in this field of research and put an end to the discrepancies observed is striking. It is therefore important to emphasize the need for studies that clearly identify the most common terms used in searching for information, something that has not been explored in previous studies, which demonstrates the importance and originality of this research.

Finding out what interests the public, through Internet information search trends, that is, how people search and “surf” the Net to obtain information, can provide valuable insights into the patterns of behaviour and habits of populations. In the health field, Eysenbach [6, 7] has defined infodemiology (information plus epidemiology) as “the science of distribution and determinants of information in an electronic medium, specifically the Internet, or in a population, with the ultimate aim to inform public health and public policy”. In other words, observing and analysing Web-based behaviour in order to examine actual human behaviour so as to predict, assess and even prevent health-related problems [8]. A well-known example of the use of Internet data in health has been the monitoring of flu outbreaks with a precision comparable to traditional methodologies [9].

An outstanding source of relevant data is Google Trends (GT), a free online search tool, which provides information on Internet searches from 2004 onwards and also geospatial and temporal patterns of search volumes for user-specified terms [10]. It has already been used to explore and monitor several health phenomena [11].

Thus, it would be interesting to know the searches that the population carries out on medications and their possible effects and problems, providing information on the frequency and appearance of possible adverse effects associated with the medication. One of the many advantages (among other disadvantages) of “cybermedicine” [12]. Furthermore, this information can be very useful for health authorities to understand the use of drugs, whether legal or not, as well as the increase or decrease in population interest [13].

Furthermore, infodemiology has been used to explore a wide range of topics in the health field and has proven to be particularly useful in the early detection of health events such as diabetes [14]. In addition, GT has also been shown to be very useful when it comes to studying population behaviours, and there are studies in the field of occupational health [15], home care [16], sexually transmitted diseases [17], as well as public health monitoring of disease outbreaks [11], among other research fields [18].

In drug studies, the work of Hanna et al. [19], showed that searches related to antibiotics revealed a high level of interest in what antibiotics are and also the appropriateness of concomitant alcohol consumption. Another study recommended that Google Trends be leveraged as a complementary infoveillance tool by government agencies to monitor and predict vaccine adherence [20]. Grandieri et al. [21], analyzed the relationship between people’s interest in medication adherence, health literacy, and self-care, demonstrating the potential of infodemiology.

Therefore, as has been demonstrated in recent years, studying information search trends can provide useful information on the behaviour and interests of the public [22]. This information could therefore be used as a guide for public health approaches to the use of medicines and the problems that may arise from them, approaches of equal utility and applicability to pharmaceutical care.

Consequently, the objective of this study was to study public interest in drug-related problems through information search trends.

## Materials and methods

### Study design

A descriptive ecological correlational study.

The methodology used in this study was based on the work of Eysenbach [6, 7], later used in health-related research [8].

### Source of information

The information search data were obtained from direct consultation of Google Trends (GT) (https://trends.google.com/trends/), by online access. The searches performed using Google as the search engine were analysed, since this tool was the market leader during the period 2010–2022, used by 83% of Internet users (Statista Research Department, 2022).

GT is a cost-free and open-access source that provides standardized statistics on Google search trends for a range of subjects. It analyses queries to determine how many searches were performed with a particular term, compared to the total number of searches made on Google with that same term in exactly the same period of time by all users. This tool excludes topics with very low search volumes (results below 1%) and duplicate searches made by the same user in a very short space of time.

### Search terms

The terms selected for the searches were obtained by direct consultation, via the Internet, of the Medical Subject Headings (MeSH) controlled language (thesaurus) databases (https://www.ncbi.nlm.nih.gov/mesh). This thesaurus is part of the project to develop a single terminology and semantic network in health, the Unified Medical Language System (UMLS) of the United States National Library of Medicine (NLM).

In principle, 5 MeSH descriptors related to DRPs were accepted for this study. Situations arising in the process of using medications that cause or may cause the occurrence of a health problem associated with the medication were considered as DRPs:

- Medication errors: errors in prescribing, dispensing or administering medication with the result that the patient fails to receive the correct drug or the indicated proper drug dosage.
- Drug overdose: accidental or deliberate use of a medication or street drug in excess of normal dosage.
- Drug-related side effects and adverse reactions: disorders that result from the intended use of pharmaceutical preparations. Included under this heading are a broad variety of chemically-induced adverse conditions due to toxicity, drug interactions and metabolic effects of pharmaceuticals.
- Contraindications, drug: a condition or factor associated with a recipient that makes the use of a specific drug improper or inadvisable.
- Drug interactions: the action of a drug that may affect the activity, metabolism or toxicity of another drug.

The conversion of MeSH descriptors to Google topics is shown in Table 1:

**Table 1:** Equivalence of MeSH descriptors to topic names in Google Trends

Medical Subject Heading (MeSH)	Google Topic
Medication errors	Medication error
Drug overdose	Drug overdose
Drug-related side effects and adverse reactions	Adverse drug reaction
Contraindications, drug	Contraindication
Drug interactions	Drug interaction

Drug-related problem: this was not included in the study since it does not exist as a topic in GT. It could only be searched for as a literal search term and would not include related topics. It would therefore introduce a significant bias into the results obtained from the search.

It is important to bear in mind that searches in GT using the term as a topic provide the results for the terms that share the same concept in any language. For example, a search for “medication error” would include those performed for “negligencia médica”, “medicina errónea”, and “erreur médicamenteuse”, among others. But, it would not include searches carried out using unusual terms, slang words or those carried out with typographical errors.

Now, GT does control the consecutive searches carried out by the same user (same IP = address of the Internet Protocol).

### Data collected and study period

All the data from the records in the study period were obtained and analysed. The study period was from 1 January 2004 (the date for which GT offers the earliest data) to 31 December 2021. The date of consultation and data collection was 14 June 2022.

### Data storage

The results obtained were downloaded in a standard format (comma-separated values) that enabled them to be subsequently stored in an Excel file. Quality control of this information was performed using double tables, correcting the possible inconsistencies by consulting the original downloaded table.

The research data, as open data, may be freely used, reused and redistributed and can be found at DOI: 10.6084/m9.figshare.21087697

### Study variables

- Relative search volume (RSV): monthly result provided by Google Trends with values standardized on a scale of 0 (a relative search volume below 1% of maximum volume) to 100 (maximum RSV). For example, an RSV of 25 represents 25% of the highest search proportion during the study period.
- Change over time: long-term behaviour or trend of the searches made on a specific subject.
- Milestone: a notable isolated event in RSV.
- Seasonality: a periodic and predictable variation in a time series with a period of a year or less.

### Data analysis

Central tendency measures were obtained to describe the quantitative variables: mean and standard deviation (σ), median, interquartile range (IQR) and maximum and minimum. The change over time of the search trends was examined with regression analysis, calculating the coefficient of determination (R^2^). To obtain the relationship between quantitative variables the Pearson correlation coefficient (R) was used. The level of significance used in all hypothesis testing was α ≤ 0.05. For this statistical analysis we used the Statistical Package for the Social Sciences (SPSS) program for Windows, version 28.0.

Seasonality was checked using the Augmented Dickey-Fuller (ADF) test. The unit root test was carried out under the null hypothesis α = 0 against the alternative hypothesis of α < 0. ADF is a type of statistical test that determines whether a unit root is present in time series data and therefore whether there is seasonality. This analysis was performed with the R program version 4.0.3 (ADF test results significant when p values >0.05).

### Ethical aspects

All data were procured from articles accepted for review. Hence, and in accord-ance with 14/2007 on biomedical research [23], approval from the Ethics and Research Committee was not required when using secondary data.

## Results

From the GT results we obtained the RSVs for the study topics: *medication error*, *drug overdose*, *adverse drug reaction*, *contraindication* and *drug interaction*. The central tendency statistics for the RSVs are shown in Table 2.

**Table 2:** Statistics for the period 1 January 2004 to 31 December 2021 on the study topics

Topic	Mean ± σ	Median	IQR	Maximum	Minimum
Medication error	0.80 ± 0.03	1	0	1	0
Drug overdose	56.25 ± 0.65	56	15	100	38
Adverse drug reaction	21.86 ± 0.56	21	8	64	12
Contraindication	44.52 ± 0.94	45	23	86	22
Drug interaction	12.67 ± 0.22	11	4	23	9

**Fig. 1:**
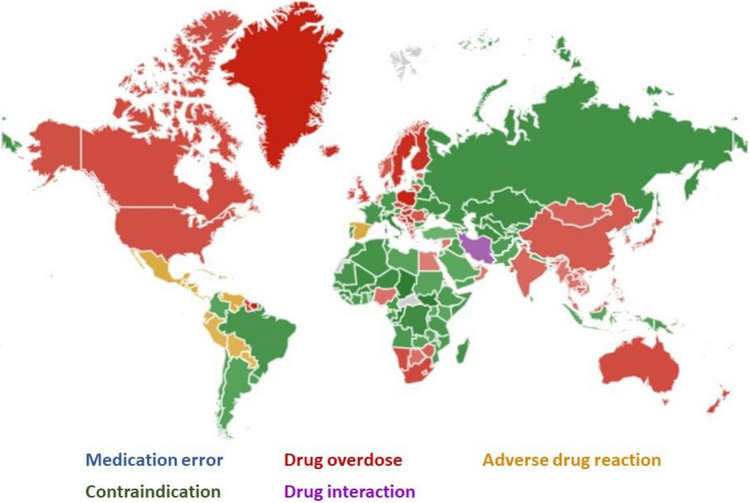
Comparative breakdown by country of the overall results, obtained from Google Trends, for the topics *medication error*, *drug overdose*, *adverse drug reaction*, *contraindication* and *drug interaction* (from 1 January 2004 to 31 December 2021)

From the data and the image provided by the GT site, we obtained the overall RSVs and the most searched topic (main interest) by country; see Fig. 1 (the intensity of the colour represents the percentage of searches and grey is due to lack of data for that area).

The world map illustrated the predominance of the terms *drug overdose*, *contraindication* and *adverse drug reaction* as leading topics, confirming that the *drug overdose* topic had the highest information search statistics, particularly in the English-speaking world.

### Change over time in relative search volumes

**Fig. 2:**
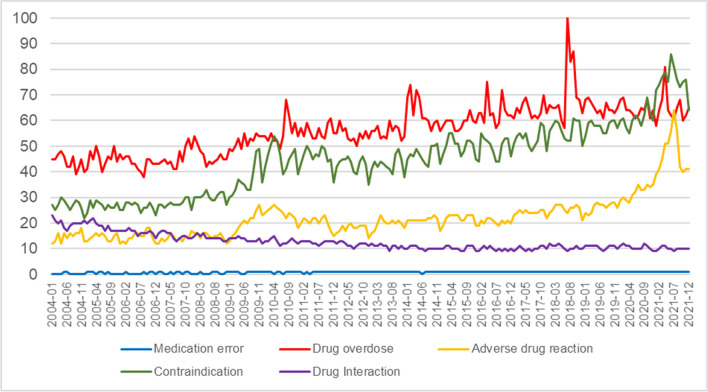
Search trends, obtained from Google Trends, for the topic’s *medication error*, *drug overdose*, *adverse drug reaction*, *contraindication* and *drug interaction* (from 1 January 2004 to 31 December 2021)

From the relative search volume data provided by GT, it was possible to construct a graph of the changes in these results over time for the terms being studied; see Fig. 2.

The respective annual RSVs exhibited a low rising linear trend for *medication error* (R^2^ = 0.33, *p* < 0.001). *drug overdose* and *adverse drug reaction* displayed an increasing exponential model (R^2^ = 0.74, *p* < 0.001 and R^2^ = 0.70, *p* < 0.001), while *contraindication* showed a very strong increasing exponential pattern (R^2^ = 0.87, *p* < 0.001). By contrast, *drug interaction* had a decreasing exponential trend (R^2^ = −0.75, *p* < 0.001).

### Milestones

The main specific event in RSV worldwide occurred in July 2018 (RSV = 100). The other topics analysed presented the following milestones: *adverse drug reaction* in July 2021 (RSV = 64), *contraindication* in June 2021 (RSV = 86) and *drug interaction* in January 2004 (RSV = 23). In the case of *medication error,* no notable milestone was found; see Fig. 2.

### Seasonality

The possible presence of seasonality — a periodic and predictable time pattern during each year of the study period — was found, in the light of the statistical analysis, in three of the topics: *adverse drug reaction*, *contraindication* and *drug interaction*.

**Fig. 3:**
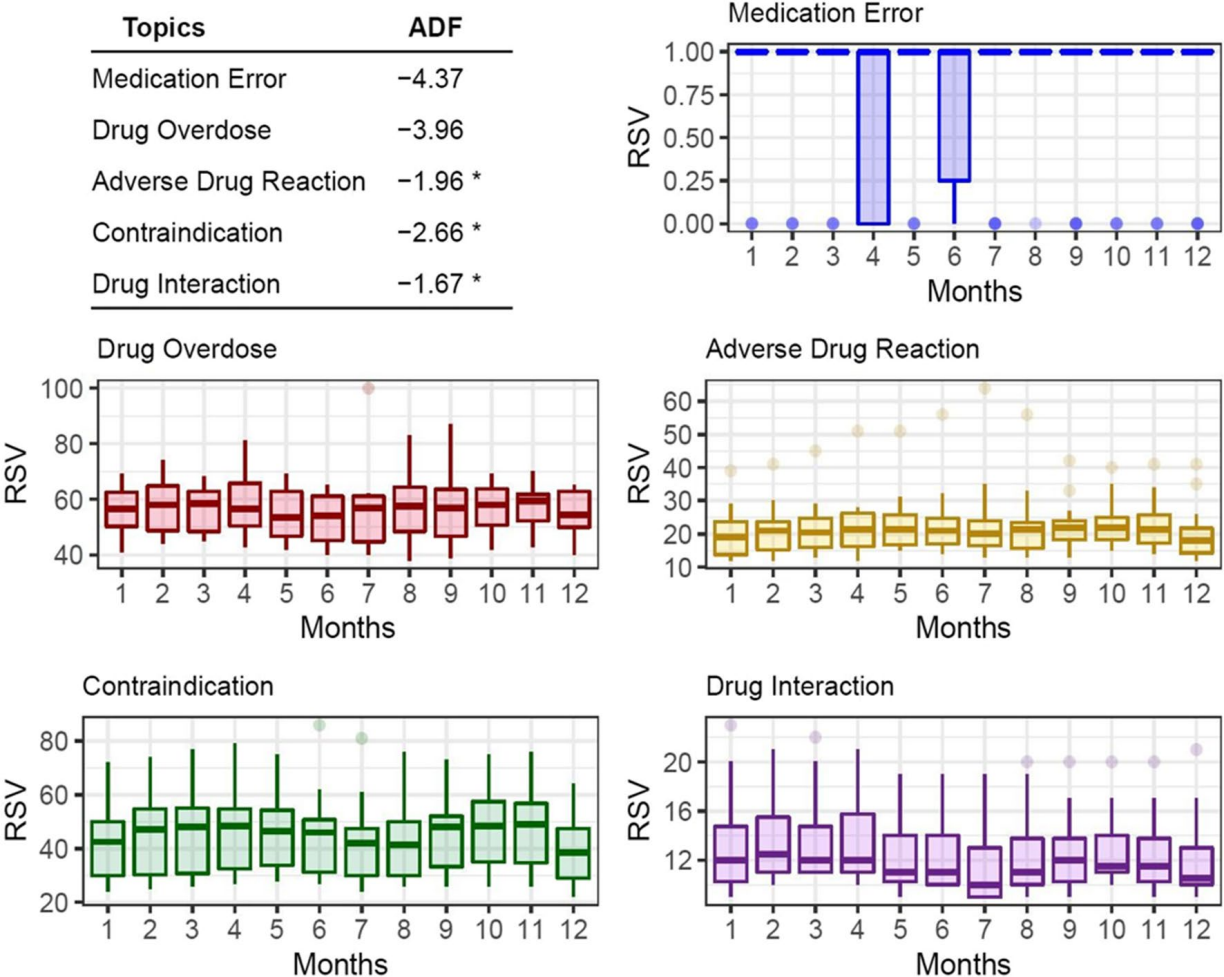
Box plots of seasonality for the *medication error*, *drug overdose*, *adverse drug reaction*, *contraindication* and *drug interaction* topics (from 1 January 2004 to 31 December 2021) grouped by month

For *adverse drug reaction*, the maximum mean RSV (mRSV) occurred in May (mRSV = 23.06 ± 1.98) and the minimum in January (mRSV = 19.61 ± 1.64). For the *contraindication* topic a maximum was observed in November (mRSV = 47.56 ± 3.39) and a minimum in December (39.94 ± 3.02), whereas for *drug interaction* a maximum was obtained in March (mRSV = 13.61 ± 0.82) and a minimum in July (mRSV = 11.50 ± 0.75); see Fig. 3.

### Relationship between the study topics

A strong direct correlation was found between *drug overdose* and *contraindication*, and a very strong one between *adverse drug reaction* and *contraindication*. The relationship observed between *drug interaction* and the other topics studied was inverse; see Table 3.

**Table 3:** Bivariate correlations between the different study topics

Topic	Medication error	Drug overdose	Adverse drug reaction	Contraindication	Drug interaction
Medication error	–	0.51*	0.36*	0.53*	−0.60*
Drug overdose	0.51*	–	0.59*	0.79*	−0.74*
Adverse drug reaction	0.36*	0.59*	–	0.89*	−0.54*
Contraindication	0.53*	0.79*	0.89*	–	−0.74*
Drug interaction	−0.60*	−0.74*	−0.54*	−0.74*	–

* The correlation is significant at the 0.05 level (bilateral)

## Discussion

In the light of the results obtained it was possible to deduce that the trends obtained from search engines can be a tool capable of monitoring the information needs of the public in real time. In this context, Anderegg & Goldsmith [24] already stated that GT was a tool recognised as a solid and valid indicator for predicting information search behaviour patterns. Jankowski & Hoffmann [25], noted that the data resulting from Google searches could be a valuable source of information for assessing the popularity of medications and the harm they cause and can even help to monitor trends in their consumption.

However, Johnson & Mehta [26] pointed out that the data being analysed are ecological and the findings might not be representative at an individual level. For example, the trends are population-based and one cannot conclude that the entire search volume is generated by individuals with drug-related problems. It is therefore important to note that the value of the RSV analysis for the proposed topics was to find out what users all over the world searched for in a particular period of time and to see how social behaviour developed.

An issue that must be made clear is that at the time when the searches were performed the topic “drug-related problem” did not exist. Consequently, the general public, like researchers, used a range of terms to locate information on the undesirable effects of medicines. This inconvenience, as already mentioned in the introduction to this article, would explain the search for a terminological consensus [1–3].

The higher result obtained for *drug overdose* may be due to the fact that a good many searches associated with suicide [27] or narcotic abuse [28] were being retrieved under this term. This is confirmed if the related queries prominently including searches on “opioid overdose” are considered. On the other hand, with “contraindication” the existence of studies related to self-medication [29] or treatment review [30] was observed. In other words, these terms are extensively used, but do not always represent the same concept.

In the comparative breakdown of the global results by country, the predominance of each topic in a particular region was clear. Although it is hazardous to associate the results with differences in Internet access between territories [17], a clear difference was observed between the interests of English-speaking countries (the United States, Canada, the United Kingdom, Australia, etc.), where the use of the “drug overdose” topic stands out, and other countries. This unity of terminological practice has already been observed in other previous studies [15, 31].

Perhaps the differences might be based on the different models of drug dispensing in the world [32], the legislative differences regulating this dispensing [33] or the avoidable harm related to medication [34]. In any case, the RSV data obtained with GT show the interest of the population, of various countries, in the subject under consideration. As has been demonstrated in previous studies [15, 35], trend trackers are a tool that can be integrated, in real time, into the monitoring of the population’s health information needs.

The search trends showed increasing values, except for the “medication error” topic, whose linear pattern demonstrated that the public had little interest in this subject or was even unaware of the term. In the case of “drug interaction”, a progressive lack of interest in searches related to this term was observed. Actually, of the five topics studied, these two seem to be more commonly used in the professional sphere: in the case of “medication error”, the public usually interprets it more as an adverse effect [36, 37], and “drug interaction” is a term little known to non-professionals [38] and generally not explained by community pharmacists [39]. Ayala-Aguirre et al. [35], have already pointed out that searches with technical words are not common in GT, and therefore most of the population tends to look for information using words in common use.

On the other hand, the “drug overdose”, “contraindication” and “adverse drug reaction” topics showed similar increases in their RSV. However, attention should be drawn to the exponential pattern presented by “adverse drug reaction”, where the highest growth was experienced from 2019. This situation was interpreted as an increased interest among the general population in adverse reactions caused by medication. It must be borne in mind, as Coleman & Pontefract [40] rightly pointed out, that adverse reactions to medicines are still a challenge for modern medical care, particularly given the growing complexity of treatments, the ageing of the population and the increase in multimorbidity.

It is true that there are differences between countries and languages, as is recognised below in the limitations of this study. Even so, one should take into account that various other factors may also exert an influence. For example, there is the great variety of healthcare systems even within the same continent; in the European Union alone there are a multitude of models with major differences in both organization and legislation [41]. Moreover, it is important to note that searches for one of the most searched terms, “drug overdose”, are concentrated in North America, which may be of interest bearing in mind that according to the United Nations Office on Drugs and Crime the United States is the leading country for consumption of illicit drugs such as cocaine, amphetamines and opioids [42].

In the study of milestones, a major peak stands out, with a maximum value in July 2018, related to the “drug overdose” topic. This conspicuous event coincides with the date when the singer Demi Lovato was hospitalized, allegedly for a heroin overdose [43], which aroused significant public interest. This observation would reinforce the idea that searches related to narcotics abuse were also being retrieved under this topic [28], though it would confirm that the incidence of milestones may be due to the appearance of news stories related to famous people or in response to specific publicity campaigns resulting in increased interest in searching for information [44].

Likewise, external factors such as epidemics that promote searches for pharmacological treatment or the need for a vaccine can influence search trends, as has been proven in the recent COVID-19 pandemic [20, 21]. On the other hand, pharmaceutical manufacturers may promote prescription drugs in the following countries only: Canada, New Zealand and United States (pharmaceutical manufacturers may not promote prescription opioid painkillers). Pharmaceutical manufacturers can promote over-the-counter medicines in a wide number of countries (searches for over-the-counter medicines were more likely to lead to commercial domains) [19]. This circumstance will also influence an increase in search trends favoured by this advertising.

The milestone trend analysis, or MTA, is a method of tracking progress in project planning. MTA charts can help research team leaders assess the health of a project and provide valuable insights about scheduling or scope for future initiatives [45].

The increases observed in the “adverse drug reaction”, July 2021, and “contraindication”, June 2021, topics could be due to the increase in DRPs caused by the COVID-19 pandemic, since there is research reflecting this [41, 46]. In the case of “drug interaction” and “medication error”, the absence of notable milestones would imply that the population has not seen any reason to perform more searches on them.

In this study seasonality was analysed as fluctuating demand for information on the topics over the course of the year. It was found that although this was present to a moderate degree in “adverse drug reaction”, “contraindication” and “drug interaction”, no very noteworthy data were obtained and they could be due to a wide range of causes. Consequently, in line with the study by Kardeş [42], more research is required to clarify the mechanisms governing this seasonality.

Seasonality is the systematic, though not necessarily regular, movement that occurs in the variables studied over the course of the year, due to changes in the characteristics of the various calendar periods. It is important to study it, as the results may influence decision-making [47] or even the implementation of preventive measures related to specific healthcare problems [48].

The existence of epidemiological behaviour in the information searches under study was not confirmed, since no sawtooth pattern at constant time intervals was observed in its graphic progression, as occurs in other health-related subjects [9]. These results rule out the possibility that the searches analysed behave in a regular fashion related to the time of year. In any case, the graphic pattern of the search trends is a visual means that enables us to recognize the connection between the alternating sections of dialogue in a colloquial conversation (whether verbal, written or digital) and provides a strong cause-effect relationship between the events studied and the need for more information [49].

When analysing the relationship between the various topics, the very strong relationship between “adverse drug reaction” and “contraindication” must be emphasized. The association may be due to the population searching for DRPs without having a clear idea of the difference between one term and another [50], though this confusion may result from the lack of clear information from the health authorities [51], which would represent a clear argument for greater international harmonization of DRPs. There is still an obvious need to adopt a term that will clearly identify the negative results associated with the use of medications and distinguish them from other process elements [52].

However, the divergence in behaviour between the most searched topics (“drug overdose”, “contraindication” and “adverse drug reaction”) and those that aroused less public interest (“medication error” and “drug interaction”) is clear. Perhaps, as has already been suggested, it is because the latter two terms are more specialized and therefore less often used by the general population.

### Possible limitations of the study

It is important to note that this study did not aim to determine the real results of DRPs. The value of the RSV analysis for the proposed topics lay in finding out what user searches were performed, worldwide, in a particular time period and to see how social behaviour evolves.

Furthermore, the reasons for searching for the various topics may not always be the same, nor can it necessarily be assumed that they will all be searched for in a single operation. This research study was based on Google and did not consider other search engines. However, Google headed the ranking of search engines with the highest number of users in both 2021 and 2022, with a worldwide market share of over 92% [53].

An important point is that GT provides relative values, but not absolute frequencies (total number of searches), which reduces the scope for more real prognosis and statistical analyses. Moreover, greater transparency would be desirable, since there is no information on the specific methods that Google uses to forecast trends; these have not been divulged by the company [54].

In addition, the lack of data regarding the geographic location of the users is a limitation in interpreting engagement.

It is accepted that the searches carried out will not include those carried out with unusual terms, words used in slang and those searches carried out with typographical errors.

Furthermore, it must be borne in mind that Google uses context to improve the results. Some examples of context are location (the most significant results for a particular search may condition how people search), language (results will be positioned depending on the language in which the search is performed), type of device (results are displayed according to the type of screen, distinguishing, for example, between mobile devices and computers) and related results.

Although “big data” such as that collected by GT may be of much value, it should be interpreted with caution as it is not possible to accurately define the population contributing to the data sample. One cannot be sure whether an individual is seeking information because they have the problem or just out of curiosity [55].

Finally, it must be acknowledged that this study is limited to the “connected world”, and there will therefore be a bias regarding the results that can be derived from the behavioural patterns of the population. Moreover, as Cervellin et al. suggest [22], the results obtained with this tool may be influenced by media coverage.

### Conclusions

The greatest public interest was found in the *drug overdose* and *contraindication* topics, and at the same time it was these that showed the largest upward trend, though the seasonality study did not produce any very significant results, nor did it demonstrate epidemiological behaviour in information searches.

The main milestone observed was due to media factors related to the consumption of narcotics.

There was a clear difference in the use of the *drug overdose* topic in English-speaking countries (the United States, Canada, the United Kingdom, Australia, etc.).

A correlation between the *adverse drug reaction* and *contraindication* topics was confirmed.

## Data Availability

The datasets generated and/or analysed during this study are available through the Google Trends tool. The research data, as open data, may be freely used, reused and redistributed and can be found at DOI: 10.6084/m9.figshare.21087697

## Abbreviations

ADF: Augmented Dickey–Fuller Test
COVID-19: Coronavirus Disease 2019
DRP: Drug-Related Problems
GT: Google Trends
IQR: Interquartile Range
MeSH: Medical Subject Headings
mRSV: maximum mean RSV
MTA: Milestone Trend Analysis
NLM: United States National Library of Medicine
UMLS: Unified Medical Language System
RSV: Relative Search Volume
SPSS: Statistical Package for the Social Sciences
